# Circulating Adipocyte Fatty Acid Binding Protein (FABP4) Levels Are Associated with Irisin in the Middle-Aged General Chinese Population

**DOI:** 10.1371/journal.pone.0146605

**Published:** 2016-01-11

**Authors:** Shan Zhang, Lili Yang, Peihong Chen, Hua Jin, Xinmiao Xie, Meili Yang, Ting Gao, Cheng Hu, Xuemei Yu

**Affiliations:** 1 Department of Endocrinology and Metabolism, Fengxian Central Hospital, Shanghai, China; 2 Department of Endocrinology and Metabolism, Third Clinical Medical College of Southern Medical University, Guangzhou, China; 3 Department of Endocrinology and Metabolism, Fifth Affiliated Hospital of Zunyi Medical College, Zhuhai, China; 4 Shanghai Diabetes Institute, Shanghai Key Laboratory of Diabetes Mellitus, Shanghai Clinical Center for Diabetes, Shanghai Jiao Tong University Affiliated Sixth People's Hospital, Shanghai, China; University of Catanzaro Magna Graecia, ITALY

## Abstract

**Background:**

Adipocyte fatty acid binding protein (FABP4) has been recently characterized as an adipokine that is closely associated with obesity and metabolic syndrome. Irisin, a novel myokine, activates thermogenesis by increasing the transformation of white adipocytes to brown, and it has improved glucose homeostasis in animal models. In this study, we aimed to explore the relationship between serum FABP4 and irisin in middle-aged Chinese subjects.

**Methods:**

A total of 111 normal residents (56 men and 55 women) of Fengxian District who were 40 to 60 years of age were recruited. Circulating FABP4 and irisin were determined by enzyme-linked immunosorbent assay. Anthropometric parameters, oral glucose tolerance test results, hemoglobin A1C (HbA1C), blood lipids, homeostasis model assessment of insulin resistance, homeostasis model assessment-β and body fat composition were also determined.

**Results:**

All participants were categorized by FABP4 tertiles. There were significant differences in blood pressure, body fat percentage, 2-h plasma glucose, and skeletal muscle mass among the three groups (*P*<0.05). Furthermore, FABP4 levels in the women were significantly higher than in the men (*P*<0.05). However, there was no sexual dimorphism in serum irisin (*P*>0.05). To exclude the effect of sex difference, partial correlations analysis showed that FABP4 was positively correlated with diastolic blood pressure (*P*<0.05) and body fat percentage (*P*<0.05) negatively correlated with skeletal muscle mass (*P*<0.05) and irisin (*P*<0.05), while irisin was positively correlated with HbA1c (*P*<0.05) and negatively correlated with creatinine (*P*<0.05). Multivariate regression analysis demonstrated that serum FABP4 was independently associated with skeletal muscle mass (*P*<0.001), diastolic blood pressure (*P*<0.05) and irisin (*P*<0.05) after adjustment for age, body mass index, body fat percentage, total cholesterol and HbA1C.

**Conclusions:**

Elevated FABP4 levels increase the risks of obesity-related metabolic disorders and hypertension. Serum irisin might exert antagonistic effects on FABP4 in the middle-aged Chinese population.

## Introduction

The fatty acid binding protein (FABP) family consists of intracellular lipid carriers that participate in regulating lipid transport and metabolism, and serum FABPs have been considered specific markers of tissue injury [1]. However, the adipocyte fatty acid binding protein (FABP4, also known as A-FABP and aP2), which is secreted from adipocytes and macrophages, has recently been investigated as a marker that is closely associated with obesity and metabolic syndrome [1]. Targeted disruption of mouse FABP4 genes elevated cellular free fatty acid levels and impaired lipolysis in adipocytes [2]. *In vivo*, disruption of FABP4 mainly affects two pathways, decreasing free fatty acid efflux in fat cells and increasing the consumption of glucose while inhibiting the utilization of fatty acids [3]. In macrophages, FABP4 participates in the regulation of the inflammatory response, promoting the concentration of cholesterol and the formation of foam cells [4]. In obese mice, FABP4 deletion improved insulin resistance and lipid metabolic disorders [5]. Elevated serum levels of FABP4 were associated with obesity, insulin resistance, dyslipidemia and hypertension in healthy subjects [1].

Irisin, a novel myokine and adipokine, is secreted by skeletal muscles and is induced by exercise [6,7]. Serum irisin promotes uncoupling protein 1 (UCP1) expression both *in vitro* and *in vivo*, and it activates the browning of white adipose tissue [6]. Increased serum irisin levels cause increases in energy expenditures and resistance to diet-induced insulin resistance in mice [6,7]. Additionally, Moreno-Navarrete et al. observed that serum irisin was negatively correlated with obesity and insulin resistance in patients with type 2 diabetes mellitus [8]. In patients with nonalcoholic fatty liver disease, serum irisin was significantly reduced, and triglycerides gradually decreased as irisin increased [9].

Secreted by adipocytes, FABP4 was positively associated with body mass index (BMI) and body fat percentage [10], whereas the correlation between irisin and BMI remains a controversial issue. However, the interaction of FABP4 and irisin has not been clarified. This study aimed to examine the relationships between serum FABP4 levels and irisin in a middle-aged Chinese population.

## Methods

### Ethics statement

This study was approved by the Medical Ethics Committee of the Shanghai Fengxian District Central Hospital and was performed in accordance with the principles of the Declaration of Helsinki as revised in 2000. All of the subjects provided written informed consent before they participated in this study.

### Participants

From December 2012 to September 2013, a total of 111 apparently healthy subjects (men/women: 56/55), aged 40 to 60 years old, were recruited for this study. None of the subjects was taking oral hypotensive, hypolipidemic, anti-diabetic, and/or other medications known to affect glucose or lipid metabolism. Individuals with histories of diabetes mellitus, acute or chronic inflammatory disorders, cancer, heart failure, active hepatitis/liver cirrhosis, chronic renal failure or other known major diseases were also excluded from the study.

### Clinical measurements

Anthropometric parameters were measured, and body mass index (BMI) was calculated as body weight (kilograms) divided by body height squared (square meters). Skeletal muscle and body fat percentages were measured using a human body composition analyzer (INBODY S10, Republic of Korea). Blood pressure was measured three times using a mercury sphygmomanometer with the subject in a seated, resting position, and the average value was acquired. Blood samples were obtained from the antecubital vein following an overnight fast of at least 10 h. Oral glucose tolerance testing (OGTT) was performed in all of the subjects, and blood samples were collected after 2 h. After clotting, serum was obtained from blood specimens by centrifugation and was stored in aliquots at -80°C until analysis of FABP4 and irisin. Aspartate aminotransferase (AST), alanine aminotransferase (ALT), uric acid, creatinine, blood urea nitrogen, total cholesterol, triglycerides, low-density lipoprotein cholesterol (LDL-c), high-density lipoprotein cholesterol (HDL-c), free fatty acid, fasting plasma glucose (FPG), and 2-h plasma glucose (2hPG) were measured using an automatic biochemical analyzer (Beckman DXC800, USA). Fasting insulin (FINS) levels were measured by electrochemiluminescence immunoassay (ADVIA Centaur, Germany), and hemoglobin A1C (HbA1C) was measured using high-pressure liquid chromatography (TOSOH HLC-723 G7, Japan). High-sensitivity C-reactive protein was measured by particle-enhanced immunoturbidimetric assay.

The homeostasis model assessment of insulin resistance (HOMA-IR) was calculated as FPG (mmol/L)×FINS (mU/L)/22.5. The homeostasis model assessment-β (HOMA-β) was calculated as 20×FINS (mU/L)/ (FPG [mmol/L]-3.5).

### Serum FABP4 and irisin measurements

Serum FABP4 concentrations were determined using enzyme-linked immunosorbent assay (ELISA) (catalog number RD191036200; BioVendor, Inc., Czech Republic), with intra-assay and inter-assay coefficients of variation (CVs) less than 9% and 15%, respectively. Irisin levels were determined by ELISA (catalog number EK-067-29; lot number 604494; Phoenix Pharmaceuticals, Inc., USA) with intra-assay and inter-assay CVs less than 10% and 15%, respectively. We first explored the concentration ranges of FABP4 and irisin in pre-experiments. According to the linear range in the manual, all of the samples were diluted with analysis buffer, and the ultimate concentrations were calibrated based on multiple dilutions. The diluted sample concentrations met the linear ranges of the kits.

### Statistics

All of the analyses were performed using SPSS software, version 19.0. The data are presented as means ± SDs or medians (interquartile ranges). Normally distributed variables were analyzed using one-way ANOVA, while abnormally distributed variables were analyzed using the Kruskal-Wallis test. Categorical variables were compared with the *x*^*2*^ test. To exclude the effects of sex difference, partial correlation coefficients were used to analyze the relationships of FABP4, irisin and clinical parameters after logarithmic transformation because of the abnormal distributions of the variables. To determine the contributions of several variables to FABP4, various parameters, including age, BMI, body fat percentage, diastolic blood pressure, skeletal muscle mass, total cholesterol, HbA1C and irisin were tested in multiple stepwise linear regression analysis. A two-sided value of *P*<0.05 was considered statistically significant.

## Results

The clinical and laboratory parameters of the study subjects are displayed in Table 1. All of the participants were categorized by serum FABP4 level tertiles (tertile 1: 5.20 ng/mL-13.76 ng/mL, tertile 2: 14.10 ng/mL-19.47 ng/mL, tertile 3: 19.59 ng/mL-47.03 ng/mL). Significant differences were observed in diastolic blood pressure, systolic blood pressure, body fat percentage, 2hPG, and skeletal muscle mass among the three groups (*P*<0.05). The values of systolic blood pressure and 2hPG were significantly lower in the 2^nd^ tertile compared to the 3^rd^ tertiles (*P*<0.05), but the difference between 1^st^ and 2^nd^ tertiles, showed no statistical significance (*P*>0.05). In addition, the levels of FABP4 in women were significantly higher than those in men (*P*<0.05). However, there were no statistically significant differences among the three groups in age, BMI, ALT, AST, total cholesterol, triglycerides, HDL-c, LDL-c, free fatty acids, FPG, HbA1c, high sensitivity C-reactive protein, HOMA-IR, HOMA-β, creatinine or uric acid. Furthermore, with increased levels of FABP4, there were no observable changes in serum irisin levels. Circulating FABP4 concentrations were not altered in overweight/obese participants (BMI ≥25 kg/m^2^), compared with non-obese subjects (BMI < 25 kg/m^2^). Moreover, there were no sexual dimorphism in serum irisin (*P>*0.05).

**Table 1:** Clinical characteristics of the study participants.

Variables	Tertile 1	Tertile 2	Tertile 3	*P* value
**FABP4 (ng/ml)**	10.28 (8.15, 13.21)	16.44 (14.98, 17.81)	24.93 (22.91, 32.39)	<0.001*
**Age (years)**	50.62±6.36	50.54±6.49	53.30±3.99	0.067
**Male/Female (n)**	(25/12)	(21/16)	(10/27)	0.023*
**Body Mass Index (kg/m**^**2**^**)**	24.18±2.58	24.34±3.34	25.57±3.12	0.110
**Body Fat Percentage (%)**	28.09±5.83	31.08±7.08	35.62±4.67	<0.001*
**Muscle Mass (kg)**	27 (23.70, 29.50)	22.35 (20.05, 28.33)	20.7 (19.00, 24.40)	0.005*
**Systolic Blood Pressure (mm Hg)**	127.76±18.43	123.51±15.20	133.71±14.25	0.030*
**Diastolic Blood Pressure (mm Hg)**	80 (70, 90)	80 (71, 90)	90 (80, 90)	0.013*
**ALT (IU/L)**	31 (20.50, 48.00)	26 (20.50, 34.00)	23 (18.00, 34.00)	0.277
**AST (IU/L)**	24 (19.25, 32.75)	23 (20, 26)	21 (18, 28)	0.267
**Total Cholesterol (mmol/L)**	5.10±1.17	5.34±1.14	5.47±1.23	0.386
**Triglycerides (mmol/L)**	1.29 (0.95, 2.47)	1.3 (1.00, 1.95)	1.51 (1.00, 2.16)	0.741
**HDL-c (mmol/L)**	1.28±0.28	1.31±0.26	1.32±0.26	0.763
**LDL-c (mmol/L)**	2.95±0.87	3.13±0.74	3.17±0.89	0.476
**Free Fatty Acids (mmol/L)**	0.48±0.28	0.41±0.19	0.49±0.24	0.297
**FPG (mmol/L)**	5.60 (5.20, 7.35)	5.5 (4.85, 6.45)	6.10 (5.15, 7.40)	0.245
**2hPG (mmol/L)**	11.70 (5.75, 14.85)	6.10 (5.30, 12.7)	12.00 (6.95, 15.75)	0.022*
**HbA1c (%)**	5.70 (5.35, 6.65)	5.60 (5.20, 6.05)	6.00 (5.45, 6.80)	0.098
**High-sensitivity C-reactive Protein (mg/L)**	1.35 (0.60, 3.65)	1.50 (0.80, 4.40)	1.40 (0.60, 6.10)	0.928
**HOMA-IR**	2.23 (1.22, 2.90)	1.92 (1.26, 3.15)	2.56 (1.39, 3.27)	0.317
**HOMA-β**	54.97 (40.18, 92.92)	67.30 (45.25, 101.35)	75.83 (33.32, 111.40)	0.560
**Creatinine (μmol/L)**	61.84±15.67	60.05±13.77	57.89±11.86	0.474
**Uric Acid (μmol/L)**	296.95±77.48	271.95±87.70	276.54±69.89	0.351
**Irisin (ng/ml)**	263.00 (214.70, 310.20)	236.90 (193.90, 340.40)	232.90 (198.10, 273.20)	0.191

Date are presented as the mean ± SD or median (interquartile range).

*, *p* < 0.05.

ALT: alanine transaminase; AST: aspartate transaminase; HDL-c: high-density lipoprotein cholesterol; LDL-c: low-density lipoprotein cholesterol; FPG: fasting plasma glucose; 2hPG: 2-h plasma glucose; HbA1c: hemoglobin A1C; HOMA-IR: homeostasis model assessment of insulin resistance; HOMA-β: homeostasis model assessment-β; FABP4: adipocyte fatty acid-binding protein

In bivariate correlation analysis, FABP4 concentrations were associated with sex (*r* = 0.348, *P*<0.001). To exclude the effects of sex difference, partial correlation analysis were used to analyze the relationship between circulating FABP4 levels and various clinical characteristics in all of the subjects. Partial correlation analysis showed that serum FABP4 concentrations were positively correlated with diastolic blood pressure (*r* = 0.270, *P* = 0.005) and body fat percentage (*r* = 0.251, *P* = 0.012) and negatively correlated with skeletal muscle mass (*r* = -0.335, *P* = 0.001) and irisin (*r* = -0.203, *P* = 0.033) in all of the subjects after adjusting for sex. Moreover, serum irisin levels were positively correlated with HbA1c (*r* = 0.190, *P* = 0.047) and negatively correlated with creatinine (*r* = -0.291, *P* = 0.002) after adjusting for sex. Surprisingly, an inverse associated between FABP4 levels and irisin was observed. However, we found that FABP4 had no significant correlations with age, total cholesterol, fasting glucose or HOMA-IR. The correlation coefficients of clinical parameters associated with circulating FABP4 and irisin are presented in Table 2.

**Table 2:** Correlation analysis of variables associated with circulating FABP4 and irisin levels.

	FABP4 (sex adjusted)		Irisin (sex adjusted)	
Variables	*r*	*P* value	*r*	*P* value
**Age (years)**	0.170	0.076	0.035	0.715
**Body Mass Index (kg/m**^**2**^**)**	0.167	0.085	0.034	0.729
**Body Fat percentage (%)**	0.251	0.012#	-0.008	0.937
Muscle mass* (kg)	-0.335	0.001#	-0.041	0.684
**Systolic Blood Pressure (mm Hg)**	0.164	0.090	0.096	0.321
Diastolic Blood Pressure (mm Hg)*	0.270	0.005#	-0.026	0.789
ALT (IU/L)*	-0.065	0.498	0.101	0.293
AST (IU/L)*	-0.014	0.866	-0.006	0.950
**Total Cholesterol (mmol/L)**	0.069	0.477	0.126	0.188
Triglycerides (mmol/L)*	0.090	0.350	0.155	0.107
**HDL-c (mmol/L)**	-0.035	0.717	0.132	0.171
**LDL-c (mmol/L)**	0.038	0.693	0.085	0.379
**Free Fatty Acids (mmol/L)**	0.063	0.523	0.047	0.635
FPG (mmol/L)*	0.034	0.726	0.184	0.054
2hPG (mmol/L)*	0.044	0.649	0.088	0.363
HbA1c (%)*	-0.007	0.939	0.190	0.047#
High-sensitivity C-reactive Protein (mg/L)*	0.064	0.516	0.152	0.119
HOMA-IR*	0.165	0.087	0.101	0.299
HOMA-β*	0.091	0.347	-0.144	0.137
**Creatinine (μmol/L)**	0.139	0.149	-0.291	0.002#
**Uric Acid (μmol/L)**	0.008	0.934	0.047	0.626
Irisin (ng/ml)*	-0.203	0.033#	*—*	*—*
**FABP4 (ng/ml)**	***—***	***—***	-0.203	0.033#

*, Log-transformed variable

#, *p* < 0.05.

ALT: alanine transaminase; AST: aspartate transaminase; HDL-c: high-density lipoprotein cholesterol; LDL-c: low-density lipoprotein cholesterol; FPG: fasting plasma glucose; 2hPG: 2-h plasma glucose; HbA1c: hemoglobin A1C; HOMA-IR: homeostasis model assessment of insulin resistance; HOMA-β: homeostasis model assessment-β; FABP4: adipocyte fatty acid-binding protein

Further multivariate stepwise linear regression analysis demonstrated that serum FABP4 levels were independently associated with skeletal muscle mass (*β* = -0.766±0.184, *P*<0.001), diastolic blood pressure (*β* = 1.074±0.303, *P* = 0.001) and irisin (*β* = -0.212±0.103, *P* = 0.043), adjusted for age, BMI, body fat percentage, total cholesterol and HbA1C (Table 3).

**Table 3:** Multiple stepwise regression analysis showing variables independently associated with serum FABP4.

Independent variables	*β*	*P*
Irisin (ng/ml)*	-0.212	0.043
Muscle Mass (kg)*	-0.766	<0.001
Diastolic Blood Pressure (mm Hg)*	1.074	0.001
**Age (years)**	0.020	0.829
**Body Mass Index (kg/m**^**2**^**)**	0.178	0.084
HbA1c (%)*	0.014	0.879
**Total Cholesterol (mmol/L)**	0.055	0.558
**Body Fat percentage (%)**	0.171	0.113

*, Log-transformed variable

## Discussion

In recent years, metabolic syndrome, which is characterized as obesity and insulin resistance, has occupied a major place in chronic disease that threatens human health. Sufficient evidence has confirmed that the plasma biomarker FABP4 is closely associated with obesity and metabolic syndrome [10,11]. In the current study, our data showed that FABP4 levels were closely associated with diastolic blood pressure and body fat percentage. Furthermore, we found a negative association between FABP4 and irisin in all of the subjects.

Secreted by fat tissue, the cytokine FABP4 plays a key role in obesity. Consistent with previous findings, our results demonstrated that serum FABP4 levels were positively correlated with body fat percentage [10,11,12]. Recent studies have confirmed that FABP4 levels were positively associated with metabolic risk factors, such as obesity, insulin resistance, dyslipidemia and the heart failure marker NT-pro BNP [1,11,12]. In obese mice treated with an FABP4 inhibitor, the phosphorylation of insulin receptor and AKT in adipose tissue was significantly increased compared with vehicle controls, demonstrating increased insulin sensitivity [13]. Therefore, deficiency in serum FABP4 might improve the disorders associated with obesity. In addition, similar to Ishimura et al. [1] and Bao et al. [12], our study also revealed that serum FABP4 levels were significantly higher in women than in men. The larger amount of body fat in women than men might have contributed to this sexual dimorphism.

The relationships between FABP4 and clinical metabolic factors have been shown in various studies. Bao et al. [12] found in Chinese subjects undergoing coronary angiography that circulating FABP4 was positively associated with HbA1c and HOMA-IR. Our present study proved that there were no significant correlations of FABP4 with HOMA-IR, total cholesterol, or HbA1c in the middle-aged general Chinese population. Differences in age, BMI and glycometabolic state might have had certain influences on the results. In our study, the age of the subjects varied from 40 to 60 years old, and none of them had histories of diabetes mellitus or hyperlipidemia.

As an adipokine, FABP4 is also closely associated with hypertension. Ota et al. observed that serum FABP4 levels were significantly higher in non-treated essential hypertensives than normotensives, and after adjustment for age, sex, and adiposity, FABP4 was an independent predictor of mean arterial pressure [14]. Our results also revealed that diastolic blood pressure was an independent factor for FABP4, indicating the potential impact of FABP4 on hypertension. The role of FABP4 in the pathogenesis of hypertension might be that FABP4 induces the transformation of the insulin-mediated endothelial nitric oxide synthase (eNOS) pathway, thus reducing NO production and endothelial dysfunction [15].

Our data showed systolic blood pressure and 2hPG were significantly lower in the 2^nd^ tertile compared to the 3^rd^ tertiles (*P*<0.05), but the difference between the 1^st^ and 2^nd^ tertiles showed no statistical significance (*P*>0.05), and in the 2^nd^ tertile of FABP4 levels, the HOMA-IR scores were lower compared to the 1^st^ and 3^rd^ tertiles, but even this difference was not statistically significant (*P*>0.05). The lower insulin resistance in the 2^nd^ tertile might have contributed to the lower 2h PG after OGTT. The higher insulin sensitivity improved the transformation of the insulin-mediated eNOS pathway, increasing NO production and resulting in lower blood pressure [15].

Recent studies have shown that the novel cytokine irisin was associated with glucose metabolic and kidney function. In accordance with Liu et al. [16], our study proved that serum irisin was positively associated with HbA1c, and the elevated irisin levels might constitute compensation for disorders of plasma glucose. Consistent with Ebert et al. [17], there was also a negative correlation between irisin and creatinine, suggesting that irisin plays an important role in the dysfunction of kidney metabolism.

The relationship between irisin and FABP4 remains unclear. Irisin is secreted by skeletal muscle and induces the browning of white adipose tissue *in vivo* [6]. In multiple murine models, increased formation of brown fat tissue was shown to exert anti-obesity effects [18]. The improvement of obesity might reduce the secretion of FABP4 by adipocytes [1]. Our study revealed that irisin and skeletal muscle mass were independently negatively associated with FABP4. Accumulating evidence has suggested that irisin ameliorates obesity, glucose/lipid metabolic disorders and insulin resistance [8,19,20], in contrast with the metabolic function of FABP4 [1,5,13]. Therefore, we speculated that serum irisin might have a slightly antagonistic effect on FABP4.

However, controversy has emerged regarding the association between irisin and BMI. Several previous studies have reported that circulating irisin was negatively associated with BMI and percentage of fat mass [8,21], and some studies have found that irisin was positively correlated with these parameters [7,22], while others have revealed no correlation between irisin and BMI [23,24]. In the current study, we showed that serum irisin levels had no significant correlations with BMI, body fat percentage or skeletal muscle mass. Metabolic factors, such as glucose, fatty acids, body composition and amount of exercise, might regulate the biosynthesis of irisin[25]. In our study, the body fat percentage of the subjects varied from 28.09±5.83 to 35.62±4.67, and the fasting plasma glucose varied from 5.5 (4.85, 6.45) to 6.10 (5.15, 7.40).

There has also been controversy regarding the association between irisin and sex. Al-Daghri. et al. revealed that serum irisin levels were higher in girls than in boys [26]. After three weeks of sprint interval training (SIT), serum irisin levels were decreased in men and increased in women, suggested a sexually dimorphic response of irisin to SIT, but the mechanism remains unclear [27]. Consistent with our study, Moraes et al. found that serum irisin levels did not differ according to sex in healthy individuals [28]. This sexual dimorphism was not observed in new one-set T2DM [21] or normally glucose tolerant subjects [29].

With the further exploration of irisin, several studies have found that immune and quantitative methods continue to generate some controversies. In the initial discovery of irisin, Bostrom et al. used western blots to demonstrate the plasma level of irisin [6]. To explore further the function of irisin, many of the subsequent studies determined mRNA levels of FNDC5 [30,31]. Moreover, several clinical studies have quantified the levels of irisin in serum using ELISA kits. The sensitivity and specificity of the antibody must be tested by quantitative western blotting for cross-reacting proteins in plasma [32]. In research into the specificity of commercially ELISA kits, Albrecht. et al. found that there were four ELISA kits that were based on polyclonal antibodies (pAbs), revealing prominent cross-reactivity with non-specific proteins in human and animal plasma [33]. However, the ELISA kits used in our study were not included among them.

There were several limitations of our study. First, it was cross-sectional; therefore, this study could not reflect the cause-effect relationship between FABP4 and irisin. Further *in vitro* studies are needed to illuminate the molecular mechanism of the interaction between FABP4 and irisin. Second, the sample size was not sufficiently large. Third, FABP4 and irisin were measured in stored samples, although the samples were sufficiently fresh. Finally, the subjects in our study were aged 40 to 60 years old. Thus, whether our findings could be extended to populations of other ages requires further investigation.

With the current study, we have shown that higher serum FABP4 levels increased the risks of obesity-related metabolic disorders and hypertension. Serum irisin levels contributed independently to circulating FABP4, and irisin might have a slightly antagonistic effect on FABP4 in the middle-aged general Chinese population. Our findings suggested that irisin might be associated with the synthesis and release of the adipokine FABP4, providing a direction for future studies to reveal the communication among cytokines related to metabolism.
